# Treatment with dapagliflozin and empagliflozin reduces concentrations of N4-acetylcytidine in plasma, a biomarker associated with vascular damage

**DOI:** 10.1186/s12933-026-03292-z

**Published:** 2026-07-24

**Authors:** Arne Gessner, Dennis Kannenkeril, Agnes Bosch, Joanna M. Harazny, Martin F. Fromm, Hannah Klinkhammer, Christian Staerk, Andreas Mayr, Roland E. Schmieder, Renke Maas

**Affiliations:** 1 Institute of Experimental and Clinical Pharmacology and Toxicology, Friedrich-Alexander-Universität Erlangen-Nürnberg, Fahrstr. 17, 91054 Erlangen, Germany; 2 FAU NeW–Research Center New Bioactive Compounds, Friedrich-Alexander-Universität Erlangen-Nürnberg, Erlangen, Germany; 3 Department of Nephrology and Hypertension, University Hospital, Erlangen, Germany; 4 Department of Human Physiology and Pathophysiology, University of Warmia and Mazury, Olsztyn, Poland; 5 Institute for Medical Biometry and Statistics, Marburg University, Marburg, Germany; 6 IUF–Leibniz Research Institute for Environmental Medicine, Düsseldorf, Germany; 7 Department of Statistics, TU Dortmund University, Dortmund, Germany

**Keywords:** SGLT2 inhibitor, Empagliflozin, Dapagliflozin, Type 2 diabetes mellitus, N4-acetylcytidine, N-acetyltransferase 10, Vascular damage, Metabolomics

## Abstract

**Background:**

Inhibitors of the sodium-glucose cotransporter 2 (SGLT2) provide cardiovascular and renal protection in both diabetic and non-diabetic patients at least in part independently of glycaemic control. Some underlying mechanisms for these clinically beneficial effects were suggested, but the picture is far from complete. In this study we aimed to apply untargeted metabolomics in order to identify new mechanistic leads.

**Methods:**

Plasma and 24-hour urine samples of 48 diabetic patients taken before and after 6 weeks of treatment from two prospective, randomized, double-blind, placebo-controlled, cross-over trials with dapagliflozin or empagliflozin were used. Additionally, plasma and urine samples of 24 diabetic patients from a prospective, randomized, controlled, parallel-arm, interventional, open-label, single centre study with either empagliflozin and linagliptin or metformin and insulin glargine for 12 weeks were used for confirmation. Changes of metabolite patterns in plasma and urine were determined by untargeted high-resolution mass spectrometry. Moreover, parameters of arterial stiffness and retinal vascular remodelling were correlated with treatment effects of the SGLT2 inhibitors on the modified nucleoside N4-acetylcytidine (ac4C), a potential biomarker for the activity of the enzyme N-acetyltransferase 10 (NAT10).

**Results:**

In accordance with previously reported results treatment with SGLT2 inhibitors led to a reduction of glucose (log_2_fc: − 0.23, adj. *p* < 0.01) and uric acid (log_2_fc: − 0.23, adj. *p* < 0.001), while 3-hydroxybutyric acid (log_2_fc: 0.84, adj. *p* < 0.001) and 3-hydroxybutyrylcarnitine (log_2_fc: 0.57, adj. *p* < 0.001) were increased in plasma. As a new finding, plasma concentrations of ac4C were reduced (log_2_fc: − 0.32, adj. *p* < 0.001) by SGLT2 inhibitors but not by the non-SGLT2 inhibiting glucose lowering treatment. Reduction of urinary concentrations of ac4C corresponded to its reduction in plasma in groups treated with the SGLT2 inhibitors. Wall thickness of retinal arterioles and central systolic blood pressure were significantly correlated to ac4C in plasma.

**Conclusions:**

Treatment with dapagliflozin and empagliflozin reduced plasma concentrations of ac4C, which was correlated with parameters for vascular health. These exploratory findings may indicate inhibition of NAT10 activity as a potential contributor to the beneficial effects on cardiovascular and renal health by SGLT2 inhibitors.

**Trial registrations:**

http://www.clinicaltrials.gov: NCT02383238, NCT02471963, NCT02752113.

**Supplementary Information:**

The online version contains supplementary material available at 10.1186/s12933-026-03292-z.

## Research Insights

**What is currently known about this topic?**

- Sodium-glucose cotransporter 2 (SGLT2) inhibitors improve cardiovascular and renal outcomes at least in part independently of glycaemic control. Potentially underlying mechanisms are not well understood. Untargeted metabolomics can be an effective tool to better understand treatment effects of SGLT2 inhibitors.

**What is the key research question?**

- Does untargeted metabolomics analysis reveal class effects of SGLT2 inhibitors that suggest so far unknown mechanisms of action for cardiorenal protection?

**What is new?**

- Untargeted metabolomics analysis identified reduction of N4-acetylcytidine in plasma as a new class effect of SGLT2 inhibitors. This could point to an inhibition of the enzyme N-acetyltransferase 10 (NAT10) as a new potential mechanism of action of SGLT2 inhibitors.

**How might this study influence clinical practice?**

- The present findings may guide future investigations into inhibition of NAT10 activity as a potential contributor to the beneficial effects of SGLT2 inhibitors.

## Background

Inhibition of the sodium-glucose cotransporter 2 (SGLT2) in proximal tubular cells e.g., with dapagliflozin or empagliflozin, is an effective treatment option in lowering blood glucose in patients with type 2 diabetes mellitus (T2DM) [1, 2]. Beyond this effect, a growing body of clinical trial evidence shows that SGLT2 inhibitors provide cardiovascular and renal protection in both diabetic and non-diabetic patients [3–7]. This implies that SGLT2 inhibitors may have additional pharmacological modes of action, beyond glycaemic control [3, 7–11]. The underlying mechanisms of cardiovascular and renal protection are not completely understood, but recent investigations suggested multiple mechanisms to be involved [12–16]. These include an elevation in ketone bodies leading to increased energy supply for cardiomyocytes or reduction of uric acid in plasma [17–19]. In addition, several pathways were associated with SGLT2 inhibition and reduced cardiovascular or renal diseases, such as stimulation of signal transducer and activator of transcription 3 (STAT3) [20, 21], reduction in the activity of nucleotide binding domain like receptor protein 3 (NLRP3) inflammasome [22, 23], reduced activation of fibroblasts by transforming growth factor (TGF)-β1 [24, 25], and modulation of the AMP-activated protein kinase/mammalian target of rapamycin complex 1/autophagy (AMPK/mTOR) signalling [26, 27]. So far, it remains unclear, if those effects occur independently from each other, and whether they are direct or indirect effects of SGLT2 inhibitor treatment or if there are mechanisms linking them.

Untargeted metabolomics has become a key tool to identify metabolic pathways affected by drugs and mechanisms of action of drugs. So far, several putative effects of SGLT2 Inhibitors beyond glucose control have already been identified by untargeted metabolomics [18, 19, 28, 29]. Yet, the common single drug focus and/or the design of the metabolomic studies conducted in this area, so far, make it difficult to attribute new signals to class effects or to differentiate effects attributable to glucose control from other effects.

In the present study, we therefore applied untargeted metabolomics to compare the effects of SGLT2 inhibitors in patients with T2DM on the metabolome in human plasma and urine across three different studies including placebo as well as an alternative glucose lowering treatment. Study 1 compared dapagliflozin vs. placebo for 6 weeks in a cross-over design, study 2 compared empagliflozin vs. placebo for 6 weeks in a cross-over design and study 3 compared empagliflozin in combination with linagliptin to a combined therapy of metformin and insulin glargine for 12 weeks in a parallel group design. This approach allowed us to single out putative class specific metabolomic effects of SGLT2 inhibitors by comparing their effects to those of other glucose lowering-treatments and to investigate correlations of changes in the metabolome with clinical parameters of arterial stiffness and retinal vascular remodelling.

## Methods

### Study design

This study analysed samples from three trials, two prospective, randomized, double-blind, placebo-controlled, cross-over trials of dapagliflozin (DAPA) and empagliflozin (EMPA) in patients with T2DM (NCT02383238 and NCT02471963, respectively), which served for discovery of metabolomic changes due to treatment with SGLT2 inhibitors. Samples from a third prospective, randomized, controlled, parallel-arm, interventional, open-label, single centre study in patients with T2DM that were randomized to receive either empagliflozin and linagliptin or metformin and insulin glargine (ELMI, NCT02752113) for 12 weeks were used for confirmation. Study protocols were approved by German drug authorities, as well as by the local ethics committee and written informed consent was obtained. All studies were conducted in accordance with the Declaration of Helsinki and the principles of “good clinical practice” guidelines.

The detailed methods and patient characteristics of the trials have been previously published [30–32]. In brief, in the DAPA and EMPA trials patients were randomized after a run-in/wash-out period to receive either once-daily oral dapagliflozin 10 mg/empagliflozin 25 mg or placebo for 6 weeks, as graphically shown in Fig. S1A and B. In the ELMI trial patients entered a run-in phase of 4 weeks if already on stable metformin medication (either 850 mg or 1000 mg bid) for at least 2 months. After 3 months on metformin monotherapy, patients were consecutively randomized (1:1) either to empagliflozin 10 mg/linagliptin 5 mg (EL group) or metformin 850 or 1000 mg/insulin glargine (dosage depending on body weight) combination therapy (MI group), as shown in Fig. S1C. In the EL group, empagliflozin was titrated up to 25 mg once daily after 14 days if fasting plasma glucose was ≥ 100 mg/dL. In the MI group, subjects initially received 2–4 units of insulin glargine per day. The dose was increased stepwise by 2 units every third day (telephone counselling) if fasting plasma glucose was not lower than 125 mg/dL until a stable dose was reached.

Blood and 24-hour urine samples were collected at baseline, after 6 weeks with placebo, and after 6 weeks with verum (DAPA and EMPA) or at baseline and after 12 weeks of treatment (ELMI). All samples were stored at − 80 °C after collection was completed and thawed just before analysis.

For metabolomic analysis, subsets of samples from the DAPA and EMPA trials were selected to have an equal number of female and male participants, as well as an equal number of samples from both randomization groups. The subgroups were representative of the respective total study population based on the demographic characteristics shown in Table 1. Cases from the ELMI trial were chosen accordingly to be suitable matches for the DAPA and EMPA subgroups.

**Table 1:** Baseline demographic characteristics

		DAPA	EMPA	ELMI	
		Treatment			
		Dapagliflozin	Empagliflozin	Empagliflozin/Linagliptin	Metformin/Insulin glargine
Total number of patients included in metabolomic analysis		24	24	24	24
Sex	Female	12	12	12	12
	Male	12	12	12	12
Age [years]		60.1 ± 8.3	60.1 ± 7.7	59.3 ± 6.8	58.5 ± 6.9
eGFR [mL/min/1.73 m²]		105.5 ± 7.0	104.8 ± 6.0	122.3 ± 20.4	119.1 ± 25.0
BMI [kg/m^2^]		29.4 ± 4.0	29.1 ± 4.5	31.2 ± 3.3	29.3 ± 2.7
HbA1c [%]		6.60 ± 0.70	6.56 ± 0.65	7.75 ± 0.75	7.65 ± 0.81
WT [µm]		14.5 ± 2.6	14.3 ± 4.3	n.a.	n.a.
cSBP [mmHg]		122.0 ± 16.9	121.4 ± 17.1	122.1 ± 5.9	122.3 ± 9.0
pSBP [mmHg]		129.9 ± 17.3	130.5 ± 13.2	133.0 ± 6.0	134.6 ± 12.2

Data are given as mean ± standard deviation; eGFR: estimated glomerular filtration rate, BMI: body mass index, HbA1c: glycated hemoglobin, WT: wall thickness of retinal arterioles, cSBP: central systolic blood pressure, pSBP: peripheral systolic blood pressure, n.a.: not applicable; eGFR calculated according to CKD-EPI creatinine equation

### Chemicals and materials

A detailed description of used chemicals and materials can be found in the Supplementary Information.

### Metabolomic analysis of plasma and urine samples

Sample preparation was performed as previously described in detail, including normalization of varying total volumes of urine samples and acquisition of raw data from plasma and urine samples [33]. In brief, data for untargeted metabolomics analysis were acquired using high resolution mass spectrometry on a Q Exactive™ Focus system (Thermo Fisher Scientific, Dreieich, Germany) coupled to liquid chromatography (Dionex Ultimate^TM^ 3000, Thermo Fisher Scientific, Dreieich, Germany) (LC-MS). Raw data was processed using Compound Discoverer 3.4 (Thermo Fisher Scientific, Dreieich, Germany). Preliminary compound annotations were made by the software and annotations for features were double-checked with entries in the Human Metabolome Database (HMDB) [34]. The annotation of selected modified nucleosides was supported by acquisition of MS^3^-fragmentation patterns on a QTRAP 6500+ system (AB Sciex, Darmstadt, Germany) coupled to liquid chromatography (LC-40 X3 system, Shimadzu, Duisburg, Germany). Respective features were detected in pooled quality control samples from the untargeted metabolomics analysis and matched by the detected m/z of molecular ions and retention time. The intensity of the intact molecular ion and MS^2^ fragments was optimized and MS^2^ fragments were further fragmented using the linear ion trap mode to achieve MS^3^ fragmentation. The fragmentation pattern was matched to in-silico fragments of the respective putative compounds using the Explorer function of Sciex OS 4 (AB Sciex, Darmstadt, Germany).

ID levels for assessing quality of annotations were assigned to features based on a classification system suggested by Schymanski et al. [35]. For the current study only features with ID level 2 or better are reported. Features with ID level 2 show very good accordance with experimental MS-data found in a respective entry in HMDB and fragmentation patterns found in MS^3^ data are in accordance with in silico fragmentation (Figs. S2 and S3). Therefore, these annotations have a high level of certainty, but still lack confirmation by a reference standard. ID level 1 annotations agree with MS- and chromatography data of a reference standard in an in-house library, hence, this means the highest certainty for identification.

### Determination of vascular parameters

To assess vascular remodelling Scanning laser Doppler flowmetry at 670 nm (Heidelberg Retina Flowmeter, Heidelberg Engineering, Germany) was performed. In this model the retinal circulation is used as a mirror of the cerebral circulation. Details of the method have been previously published [36]. In brief, outer arteriolar diameter (OD) of the retinal arterioles is measured in reflection images, and inner lumen diameter (ILD) of the retinal arterioles is assessed in perfusion images. Wall thickness (WT) is calculated using the formula OD – ILD/2.

To derive the central arterial waveform, a validated system (SphygmoCor™ System) was used and was previously described in detail [37]. The device automatically generates the central (aortic) waveform from the radial artery waveform by a validated transfer function so that central systolic and diastolic blood pressure are obtained.

### Statistics and data analysis

Combined data from the untargeted metabolomics analysis of plasma samples of the DAPA and EMPA trials were used for discovery of features changed due to treatment with an SGLT2 inhibitor. Data from untargeted metabolomics analysis of the ELMI trial was used for confirmation of effects of SGLT2 inhibitor treatment on N4-acetylcytidine (ac4C) and other modified nucleosides. In the ELMI trial one subject from the metformin/insulin glargine treatment group was excluded from downstream analysis, due to insufficient volume of plasma available for LC-MS measurement.

Median intra-individual log_2_-fold changes (log_2_fc) for features were calculated by the ratio of the peak area of a feature found after treatment with an SGLT2 inhibitor or metformin/insuline glargine as numerator compared to the peak area found after placebo treatment or at baseline, as appropriate (DAPA and EMPA), or the corresponding baseline (ELMI) as denominator. Paired t-tests for screening in the metabolomics data were performed for each feature using RStudio (2024.12.1). For every feature detected peak areas were log_10_-transformed and the corresponding values were tested using paired t-tests. To account for multiple comparisons, *p* values were adjusted using the Benjamini–Hochberg procedure. Log_2_fc are shown as the median of the observed intraindividual log_2_fc with the respective lower and upper limit of the 95% confidence interval (shown in square brackets), as appropriate.

To test whether there is a significant difference in the change of ac4C depending on the treatment in the DAPA and EMPA trials, we fitted a linear mixed model to account for the cross-over design (i.e., repeated measurements). We used the log_2_fc in ac4C from the previous time-point as outcome and treatment, sex, age, period and sequence as fixed effects and the patient ID as a random effect. In the ELMI trial the comparison of the log_2_fc from baseline to treatment between the two arms was adjusted for sex and age.

For hierarchical cluster analysis, pairwise distances of the log_2_fc for modified nucleosides found in plasma and urine were calculated using the Euclidean distance metric and hierarchical clustering was performed with complete linkage. The results were visualized as a heatmap with dendrograms using the pheatmap package in R.

For correlations between ac4C and clinical parameters Spearman correlations were calculated. For parameters that were available for all three trials (DAPA, EMPA and ELMI), only the first period of DAPA and EMPA trials were used to facilitate pooling of the data despite different study design. For statistically significant correlations with the change of ac4C we additionally used linear models with change in parameter (dependent variable) and log_2_fc in ac4C (independent variable) and corrected for sex, age, treatment, and study. When using data of the DAPA and EMPA trials only, linear mixed models were used with period and sequence as additional fixed effects and patient ID included as a random effect.

## Results

### Patients

Detailed patient characteristics of the underlying clinical trials have been previously published [30–32]. Key characteristics of the patients selected for metabolomic analysis were balanced within the DAPA/EMPA and the ELMI trial (Table 1).

### Changes in plasma after treatment with SGLT2 inhibitors

**Fig. 1:**
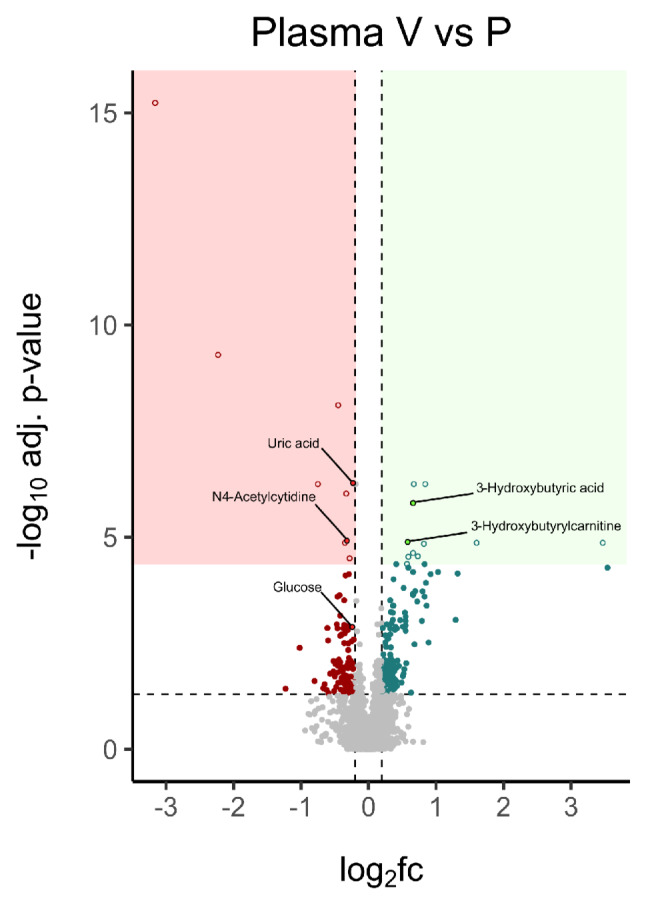
Volcano plot of untargeted metabolomics data of plasma samples after treatment with dapagliflozin and empagliflozin. The median intraindividual log_2_-fold changes (log_2_fc) in plasma after verum treatment (V) in comparison to placebo treatment (P) from the DAPA and EMPA trials are plotted against the respective *p* values from paired t-tests adjusted via Benjamini-Hochberg. Dashed lines indicate cut-off values of adjusted *p* value < 0.05 and − 0.23 < log_2_fc < 0.23. Symbols in the red and green shaded areas indicate features with the 20 lowest adjusted *p* values

Overall intraindividual changes of features detected in plasma after treatment with dapagliflozin and empagliflozin in comparison to placebo treatment are shown in Fig. 1. Out of the 20 most significantly changed features (i.e., lowest adj. *p* values), that had at least the same modulus of reduction or increase as glucose (log_2_fc: − 0.23 [− 0.36 to − 0.14], adj. *p* < 0.01), four could be unequivocally identified: 3-hydroxybutyric acid (log_2_fc: 0.84 [0.56 to 1.18], adj. *p* < 0.001) and 3-hydroxybutyrylcarnitine (log_2_fc: 0.57 [0.24 to 0.93], adj. *p* < 0.001) were increased after verum treatment in comparison to placebo treatment, while a reduction was found for uric acid (log_2_fc: − 0.23 [− 0.32 to − 0.13], adj. *p* < 0.001) and for ac4C (log_2_fc: − 0.32 [− 0.45 to − 0.11], adj. *p* < 0.001). All changes were also statistically significant if changes were calculated separately for the DAPA and EMPA trial. Table S1 shows the respective results for 3-hydroxybutyric acid, 3-hydroxybutyrylcarnitine and uric acid, while results for ac4C are shown below.

**Fig. 2:**
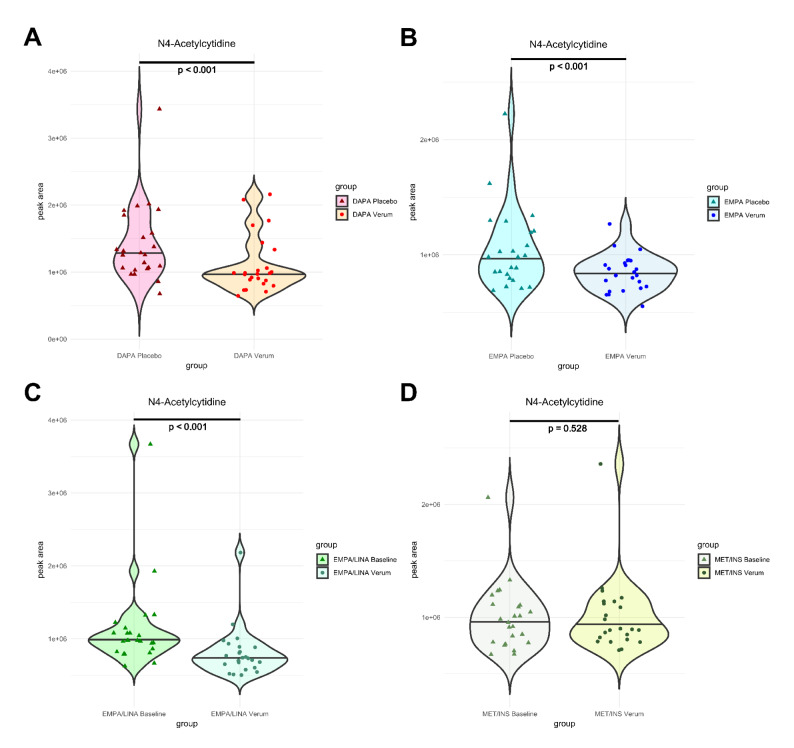
Violin plots of peak areas found for N4-acetylcytidine in plasma. Data points indicate peak areas found in plasma samples (**A**) in the DAPA trial after placebo and verum treatment, **B** in the EMPA trial after placebo and verum treatment, **C** in the ELMI trial for treatment with empagliflozin/linagliptin (EMPA/LINA) at baseline and after 12 weeks of treatment, and **D** in the ELMI trial for treatment with metformin/insulin glargine (MET/INS) at baseline and after 12 weeks of treatment. Horizontal line in the violins indicates median peak area of the respective subgroup, number on top of the figures indicates the respective *p* values from paired t-tests comparing verum vs. placebo treatment (**A**, **B**) and baseline vs. treatment (**C**, **D**)

As ac4C has not been described in this context before we calculated the changes of ac4C in plasma in the DAPA, EMPA and ELMI trials separately in order to confirm the discovery of ac4C reduction in the combined data of the DAPA and EMPA trials (Fig. 2). The reduction of ac4C was statistically significant throughout all groups that were treated with SGLT2 inhibitors with log_2_fc of − 0.39 [− 0.48 to − 0.06] after dapagliflozin, − 0.16 [− 0.44 to − 0.04] after empagliflozin and − 0.41 [− 0.62 to − 0.28] after empagliflozin/linagliptin (all *p* < 0.001), while no significant change was found after treatment with metformin/insulin glargine (log_2_fc 0.08 [− 0.07 to 0.15], *p* = 0.528).

In the combined data of the DAPA and EMPA trials, the reduction of ac4C in plasma was significantly more profound after SGLT2 inhibitor treatment than after placebo treatment (covariates-adjusted log_2_fc: − 0.42 [− 0.55 to − 0.29], *p* < 0.001) with a significant period and sequence effect in the DAPA and EMPA trials (Fig. S4). In sequence 1 (first verum, then placebo) ac4C decreased after verum in the first period (log_2_fc: − 0.32 [− 0.51 to − 0.16] vs. baseline), but then increased again after placebo in the second period (log_2_fc: 0.17 [0.01 to 0.46] vs. verum). However, there was an overall reduction of ac4C after placebo at the end of the trials compared to baseline (log_2_fc: − 0.17 [− 0.46 to − 0.01]). On the other hand, in sequence 2 (first placebo, then verum) ac4C did not substantially change in the first period after placebo (log_2_fc: − 0.03 [− 0.18 to 0.09] vs. baseline), but decreased after verum in the second period (log_2_fc: − 0.34 [− 0.52 to − 0.06] vs. placebo). The covariates-adjusted difference in log_2_fc between the two treatment arms in the ELMI trial was − 0.47 [− 0.64 to − 0.30] (*p* < 0.001).

### Changes of ac4C in plasma differ from other modified nucleosides after treatment with SGLT2 inhibitors

**Fig. 3:**
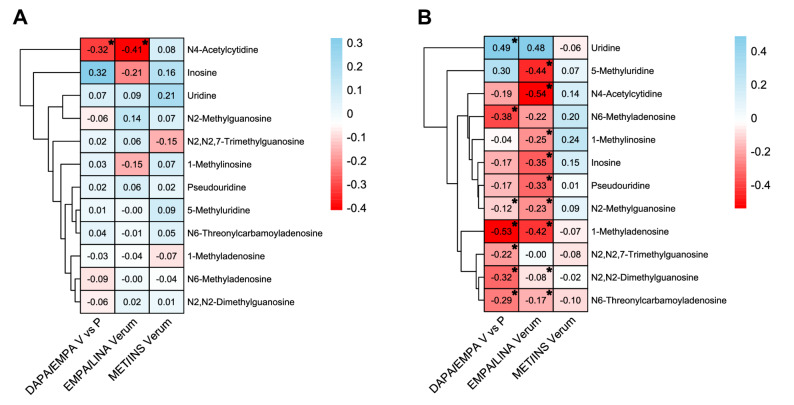
Hierarchical cluster analysis for changes of modified nucleosides in plasma and urine samples. Numbers indicate median intraindividual log_2_-fold changes found in **A** plasma and **B** urine samples of the combined DAPA and EMPA trials for verum treatment vs. placebo treatment (DAPA/EMPA V vs. P; V: verum, P: placebo), as well as for the ELMI trial for treatment with empagliflozin/linagliptin (EMPA/LINA Verum) at baseline vs. after treatment and in the ELMI trial for treatment with metformin/insulin glargine (MET/INS Verum) at baseline vs. after treatment. Distances were calculated using the Euclidean distance metric and hierarchical clustering was performed with complete linkage. * indicates log_2_-fold changes with an adjusted *p* value < 0.05

In the available metabolomic data we identified several other modified nucleosides with sufficient quality and confidence (ID level 2 for N2,N2,7-trimethylguanosine and N6-threonylcarbamoyladenosine, all others modified nucleosides shown here with ID level 1) in both plasma and urine in all trials. In plasma, ac4C was the only modified nucleoside that was significantly changed after SGLT2 inhibitor treatment, but not after treatment with metformin/insulin glargine. Hierarchical cluster analysis showed a clear distinction of ac4C from the other nucleosides (Fig. 3A). In the metabolomic data of plasma samples, there were no features to be annotated as unmodified cytidine. Two features with very low intensity may correspond to 5-methylcytidine and 2’-O-methylcytidine. However, mass spectrometric data quality did not allow annotation with sufficient confidence and they were detected in the DAPA and EMPA trials only. Both features appeared unchanged due to SGLT2 inhibitor treatment compared to placebo (log_2_fc: 0.01 and − 0.02, respectively).

In urine, significantly reduced amounts of 1-methyladenosine, N2,N2-dimethylguanosine and N6-threonylcarbamoyladenosine were found after all treatments with an SGLT2 inhibitor. However, ac4C was only significantly reduced after treatment with empagliflozin/linagliptin in the ELMI trial (Fig. 3B).

### Correlation of N4-acetylcytidine with vascular parameters

**Fig. 4:**
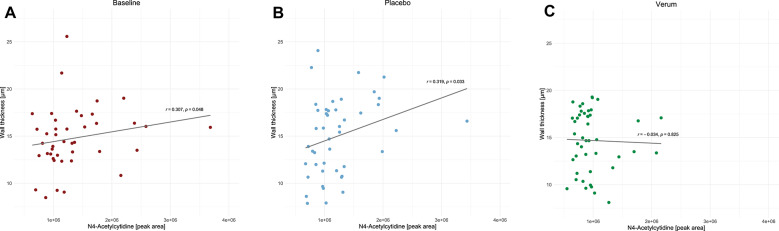
Scatter plots for correlation of N4-acetylcytidine with wall thickness of retinal arterioles. Peak areas of N4-acetylcytidine found in plasma were correlated with wall thickness of retinal arterioles in the DAPA and EMPA trials at baseline (**A**), after placebo (**B**) and after verum (**C**). Spearman correlation coefficients (*r*) and respective *p* values (*p*) are shown in the graphs

In the DAPA and EMPA trials detected peak areas of ac4C correlated significantly with wall thickness of retinal arterioles at baseline (*r* = 0.307, *p* = 0.048; *n* = 42; Fig. 4A) and after placebo (*r* = 0.319, *p* = 0.033; *n* = 45; Fig. 4B), but not after verum (*r* = − 0.034, *p* = 0.825; *n* = 42; Fig. 4C). However, there was no significant association between the change in ac4C and the change in wall thickness. In the ELMI trial the parameter retinal arteriolar wall thickness was not determined.

**Fig. 5:**
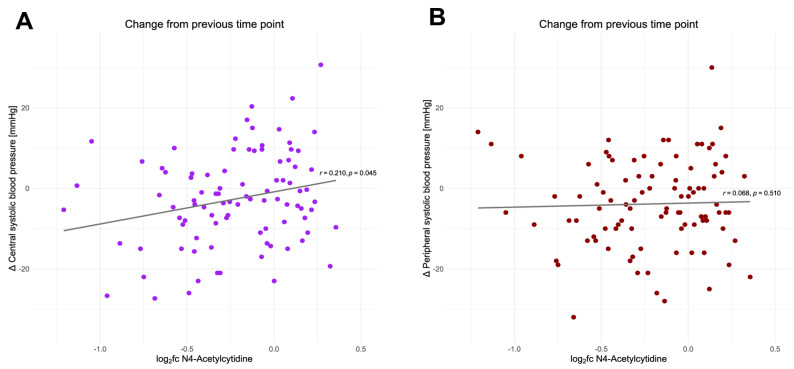
Scatter plots for correlation of N4-acetylcytidine with central and peripheral systolic blood pressure. Log_2_-fold changes (log_2_fc) of N4-acetylcytidine found in plasma were correlated with change (Δ) of central systolic blood pressure (**A**) and of peripheral systolic blood pressure (**B**) in the DAPA, EMPA and ELMI trials (all changes calculated from previous time points). Spearman correlation coefficients (*r*) and respective *p* values (*p*) are shown in the graphs. For results of regression analyses on the association adjusting for sex, age, treatment and study see results section

After treatment with an SGLT2 inhibitor, the log_2_fc of ac4C was significantly correlated with the change of central systolic blood pressure (*r* = 0.210, *p* = 0.045; *n* = 92; Fig. 5A). After correction for sex, age, treatment and study in a linear model the association remained statistically significant with β = 7.97 and *p* = 0.003. Notably, there was no statistically significant correlation between the log_2_fc of ac4C and the change of peripheral systolic blood pressure (*r* = 0.068, *p* = 0.510; *n* = 95; Fig. 5B).

## Discussion

In this study we investigated metabolomic effects of treatment with SGLT2 inhibitors and identified ac4C as a new potential biomarker correlated with putative beneficial effects on the vascular system. Furthermore, our data confirmed previously described changes of 3-hydroxybutyric acid, 3-hydroxybutyrylcarnitine and uric acid in plasma [17–19], which supports the validity of our findings. The change of ac4C was distinct from changes of other modified nucleosides and points to an inhibition of the activity of the enzyme N-acetyltransferase 10 (NAT10) by dapagliflozin or empagliflozin as the potentially underlying mechanism.

NAT10 is the only known enzyme catalysing the acetylation of cytidine to ac4C and there is no targeted process identified to reverse this process [38, 39]. This kind of mRNA modification is highly conserved in both procaryotes and eucaryotes. The acetylation leads to a stabilization of the respective mRNA transcripts, and hence prolonged expression of the respective protein [38, 39]. Because NAT10 is the enzyme responsible for the biosynthesis of ac4C and circulating ac4C levels vary across pathological conditions that were associated with NAT10 activity, the concentration of ac4C in plasma may reflect the balance between the formation of ac4C by NAT10 and its systemic elimination [38, 39]. To the best of our knowledge, so far, an effect of treatment with SGLT2 inhibitors on NAT10 activity or acetylation of cytidine was not reported yet.

In our analysis we detected a highly significant reduction of ac4C in plasma in patients treated with dapagliflozin or empagliflozin. The fold-change of the observed reduction was comparable to that of glucose. This reduction appeared to be specific and distinct from other modified nucleosides. Two features that could putatively be annotated as 5-methylcytidine and 2’-O-methylcytidine did not change in plasma. Urinary concentrations of ac4C were not increased, but decreased, therefore the reduction in plasma cannot be attributed to an inhibition of tubular reabsorption, unlike the observed reduction of glucose in plasma. This indicates that the reduction in plasma is neither a general effect of altered RNA modifications, nor the result of abnormal renal excretion of ac4C. Consequently, it can be concluded that the biosynthesis of ac4C itself must be altered.

NAT10 has been implicated as a modulating factor in the development and progression of various diseases, among them malignancies, inflammatory diseases and cardiovascular remodelling [38–41]. Furthermore, this enzyme may play a role in the regulation of several pathways related to cardiovascular health [42–45]. Two recent publications reported that ac4C-mediated transcriptomic regulation had direct effects on vascular and cardiac remodelling [40, 41]. In those studies, high activity of NAT10 led to a higher rate of cardiac hypertrophy or post-injury neointima formation and the authors suggested that NAT10 could be a promising pharmacological target. Another study however, reported a higher rate of cardiomyocyte apoptosis and resulting heart insufficiency upon knockout of NAT10 in a mouse model [46]. The authors concluded, that partial loss of NAT10 activity decreases the capacity for hypertrophic growth, whereas a (near)-complete loss of NAT10 reduces cardiomyocyte survival. Together with our present results, these studies imply, that some of the beneficial cardiovascular effects of SGLT2 inhibitor treatment may be linked to NAT10 activity.

In our study, we found a correlation between ac4C plasma concentrations and retinal arteriolar wall thickness, a structural parameter of arteriolar remodelling, at baseline and after placebo treatment, but not after verum treatment. This observation is supportive of the previously reported ac4C-mediated vascular remodelling hypothesis [40, 41]. Previously, we did not report changes in retinal arteriolar remodelling with SGLT2 inhibition [30]. Accordingly, when looking at the changes of ac4C and retinal arteriolar wall thickness in SGLT2 inhibitor treated patients we did not find a significant correlation. This could be explained by the short 6-week treatment in the DAPA and EMPA trial. In the DAPA, EMPA and ELMI trials, we have described changes in central systolic blood pressure with SGLT2 inhibitor treatment [30–32]. Presently, in the combined data of these trials we found a significant correlation between the change of ac4C in plasma and systolic central blood pressure, an important parameter of vascular aging and vascular stiffness. Interestingly, there was no significant correlation with the change of peripheral systolic blood pressure, which suggests that ac4C is related to vascular stiffness, but not to the hemodynamic parameter systolic blood pressure. This finding may point to a connection of dapagliflozin or empagliflozin treatment effects beneficial to cardiovascular health and ac4C-mediated mRNA modifications, but further research in larger samples sets is necessary in this respect.

We observed a reduction of ac4C in plasma i.e., in the systemic circulation. Therefore, we cannot make inferences regarding the acetylation of cytidine residues in certain tissues. The primary pharmacological effect of dapagliflozin or empagliflozin occurs in proximal tubular cells, where SGLT2 is expressed. These cells experience an intense reduction of available glucose, due to the effective inhibition of reabsorption from urine, which is likely disproportionally higher than the overall reduction of glucose in plasma. Therefore, their metabolic response to reduced glucose availability would be more drastic than in other tissues. Hence, it is possible that this pronounced tissue-specific reduction in glucose could result in metabolomic changes that are detectable in plasma. It is known, that SGLT2 inhibitors can have off-target effects in tissues like cardiomyocytes or macrophages [47–49]. In order to assess whether any of these effects are associated with the observed reduction of ac4C and whether it is attributable to certain tissues or rather a systemic effect, further tissue-specific analyses of the ac4C response to a SGLT2 inhibitor treatment are necessary.

Our current data do not allow to distinguish direct from indirect effects of SGLT2 inhibitors on ac4C. However, several of our observations favour an indirect effect. Regarding the respective mechanism, the results from the ELMI trial make it unlikely that the reduction of ac4C is a secondary effect of glucose reduction, since the MI group had a more pronounced reduction of fasting plasma glucose and HbA1c than the EL group, but ac4C was only reduced in the latter group. Since there was a sequence effect on ac4C in the DAPA and EMPA trials i.e., ac4C levels in the subgroups treated with verum in the first period and placebo in the second period, did not completely return to baseline levels within 7 weeks (1 week wash-out and 6 weeks of placebo treatment), a direct inhibition of NAT10 by dapagliflozin or empagliflozin appears less likely and may point towards more profound adaptation processes specific to SGLT2 inhibition leading to a reduction of ac4C. Further studies are necessary to investigate if the observed association of a reduction of ac4C and an increase in vascular health measures in the groups treated with an SGLT2 inhibitor are directly or indirectly attributable to a reduced NAT10 activity or other mechanisms. In this context, a comparison of the metabolomic effects of dapagliflozin or empagliflozin with the NAT10 inhibitor remodelin could be a useful approach.

Some limitations of the present study need to be taken into consideration. The untargeted metabolomics approach we used limits the number of samples that can be analysed with reasonable resources and in a reasonable amount of time. However, we found consistent results in three independent clinical trials. In the DAPA and EMPA trials the time under verum treatment was 6 weeks and in the ELMI trial the time was 12 weeks. This does not allow conclusions about potential long-term effects or counter-regulations for longer treatment periods with SGLT2 inhibitors. Long-term observations may be useful to further assess the herein reported associations of ac4C with clinical parameters or to find additional correlations of ac4C with clinical outcomes. Further prospective studies with larger samples sizes and absolute quantification of ac4C are needed to validate the observed correlations. Data from additional patients under SGLT2 inhibitor treatment could add statistical power, while absolute concentrations of ac4C would facilitate cross-study comparisons e.g., using data from the general population and/or healthy subjects. Furthermore, additional data from (epi-)transcriptomic analyses or functional experiments with respect to ac4C and vascular phenotypes would be helpful to test our herein described hypotheses, especially regarding NAT10 inhibition as a potential underlying mechanism. We investigated the metabolome in diabetes patients, however other populations may have varying treatment effects. Nevertheless, our observations appear to be independent of the reduction of glucose in plasma. As we found similar reductions for both dapagliflozin and empagliflozin with a significant sequence effect in two cross-over clinical trials, our reported results support that these are class effects of SGLT2 inhibitors. Established cardiovascular disease besides T2DM was not an exclusion criterion in the original clinical trials. However, this reflects the real-world situation in patients with T2DM, who are at very high risk for other cardiovascular diseases. Due to the small sample size in the current study important potential confounding effects have to be considered. These are in particular: different T2DM duration, other cardiovascular diseases and background medication. However, patients did not have any acute cardiovascular event during the last 3 months (DAPA) and 6 months (EMPA and ELMI) before inclusion. Patients with uncontrolled hypertension and reduced renal function (eGFR < 60 mL/min/1.73 m²), were also excluded from the clinical trials. The included patients did not suffer from any new cardiovascular event during their study participation and medication for other diseases was kept stable. Thus, we believe that the effect of existing cardiovascular disease besides T2DM could be regarded as a constant background-noise effect, which is unlikely to have distorted the described effects of SGLT2 inhibitors.

## Conclusions

Untargeted metabolome analyses across three clinical trials identified a reduction of ac4C plasma concentrations as a new effect of the SGLT2 inhibitors dapagliflozin and empagliflozin, with indirect effects on the activity of the enzyme NAT10 as a possible mechanism. This enzyme was previously reported to be important for vascular remodelling and we found correlations with parameters for vascular health and ac4C in the present study. Therefore, these exploratory findings may guide future investigations into inhibition of NAT10 activity as a potential contributor to the beneficial effects of SGLT2 inhibitors on cardiovascular and renal outcomes.

## Supplementary Information

Below is the link to the electronic supplementary material.

Supplementary Material 1

## Data Availability

The datasets used and analysed during the current study are available from the corresponding author on reasonable request.

## Abbreviations

ac4C: N4-Acetylcytidine
AMPK/mTOR: AMP-activated protein kinase/mammalian target of rapamycin complex 1/autophagy
DAPA: Prospective, randomized, double-blind, placebo-controlled, cross-over trial of dapagliflozin (NCT02383238)
ELMI: Prospective, randomized, controlled, parallel-arm, interventional, open-label, single centre study in patients with T2DM being randomized to receive either empagliflozin and linagliptin or metformin and insulin glargine (NCT02752113)
EMPA: Prospective, randomized, double-blind, placebo-controlled, cross-over trial of empagliflozin (NCT02471963)
HMDB: Human metabolome database
ILD: Inner lumen diameter of retinal arterioles
LC-MS: Mass spectrometry coupled to liquid chromatography
Log_2_fc: Log_2_-fold changes
NAT10: N-Acetyltransferase 10
NLRP3: Nucleotide binding domain like receptor protein 3
OD: Outer arteriolar diameter of retinal arterioles
QC: Quality control
SGLT2: Sodium-glucose cotransporter 2
STAT3: Signal transducer and activator of transcription 3
T2DM: Type 2 diabetes mellitus
TGF-β1: Transforming growth factor β1
WT: Wall thickness of retinal arterioles
