# Supplementary material for: Treatment with dapagliflozin and empagliflozin reduces concentrations of N4-acetylcytidine in plasma, a biomarker associated with vascular damage
Source: Cardiovasc Diabetol. 2026 Jul 24;25:210. doi: 10.1186/s12933-026-03292-z (PMC13401296; doi:10.1186/s12933-026-03292-z)
Supplement: Supplementary file 1 — Supplementary Material 1 [file 12933_2026_3292_MOESM1_ESM.docx]

**SUPPLEMENTARY INFORMATION**

**Treatment with dapagliflozin and empagliflozin reduces concentrations of N4-acetylcytidine in plasma, a biomarker associated with vascular damage**

Arne Gessner^1,2,#^, Dennis Kannenkeril^3,#^, Agnes Bosch^3^, Joanna M. Harazny^3,4^,

Martin F. Fromm^1,2^, Hannah Klinkhammer^5^, Christian Staerk^6,7^, Andreas Mayr^5^, Roland E. Schmieder^3^, and Renke Maas^1,2^

^1^Institute of Experimental and Clinical Pharmacology and Toxicology, Friedrich-Alexander-Universität Erlangen-Nürnberg, Erlangen, Germany

^2^FAU NeW – Research Center New Bioactive Compounds, Friedrich-Alexander-Universität Erlangen-Nürnberg, Erlangen, Germany

^3^Department of Nephrology and Hypertension, University Hospital Erlangen, Germany

^4^Department of Human Physiology and Pathophysiology, University of Warmia and Mazury, Olsztyn, Poland

^5^Institute for Medical Biometry and Statistics, Marburg University, Marburg, Germany

^6^IUF – Leibniz Research Institute for Environmental Medicine, Düsseldorf, Germany

^7^Department of Statistics, TU Dortmund University, Dortmund, Germany

^#^equal contribution

Address for correspondence: A. Gessner, Institute of Experimental and Clinical Pharmacology and Toxicology, Friedrich-Alexander-Universität Erlangen-Nürnberg, Fahrstr. 17, 91054 Erlangen, Germany. Tel: +49 9131 85 22869; Fax: +49 9131 85 22773; E-mail: arne.gessner@fau.de

**Chemicals and Materials**

*Components for LC-MS analysis*

Acetonitrile, methanol, water and formic acid (all LC-MS-grade) were purchased from VWR chemicals (Darmstadt, Germany). Ammonium formate (LC-MS-grade) was purchased from Sigma-Aldrich (Darmstadt, Germany). Pierce LTQ Velos ESI Positive Ion Calibration Solution and Pierce ESI Negative Ion Calibration Solution were purchased from Thermo Fisher Scientific (Dreieich, Germany).

*Recovery standards, internal standards and reference standards for metabolomic analysis*

Tridecanoic acid, DL-2-fluorophenylglycine, DL-4-chlorophenylalanine, phenolphthalein β-D glucuronide, and imipramine were purchased from Sigma-Aldrich (Darmstadt, Germany). [^2^H_24_]-tetradecanedioic acid was from EQ Laboratories (Augsburg, Germany). [^2^H_5_]‑tryptophan was from Santa Cruz Biotechnology (Heidelberg, Germany). [^2^H_3_]‑pravastatin was from Toronto Research Chemicals (Toronto, ON, Canada). All reference substances for identification of features in metabolomics analyses were from Sigma-Aldrich (Darmstadt, Germany).


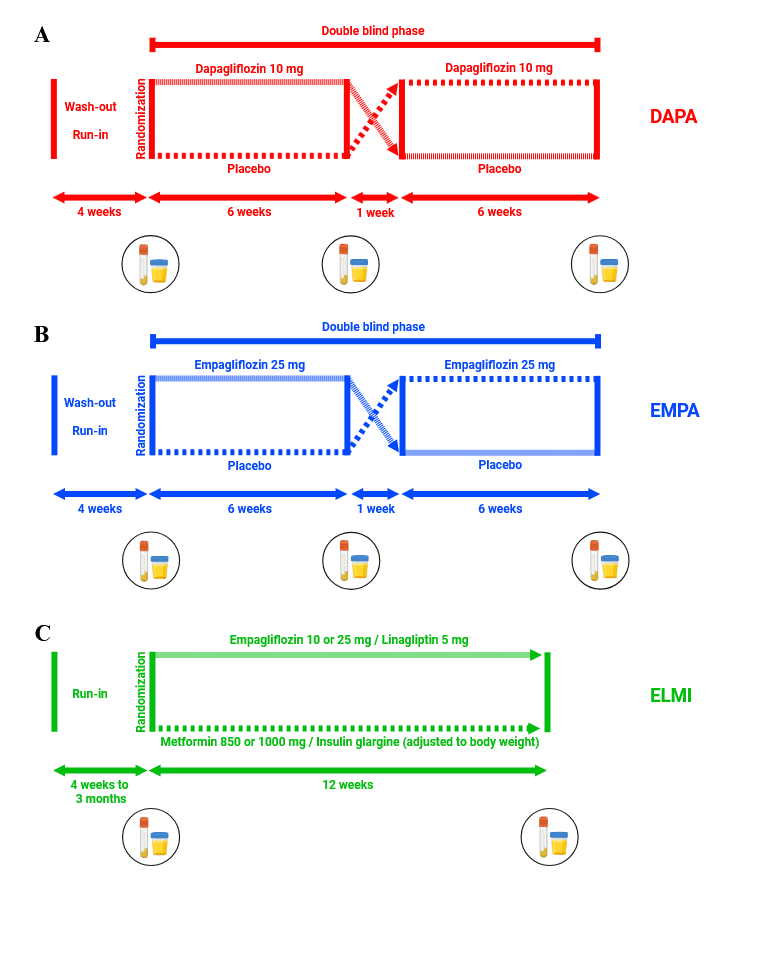


**Figure S1:** Design of the clinical trials with (A) dapagliflozin (DAPA), (B) empagliflozin (EMPA) and (C) empagliflozin / linagliptin or metformin / insulin glargine (ELMI). Plasma and urine samples were taken at the indicated time points


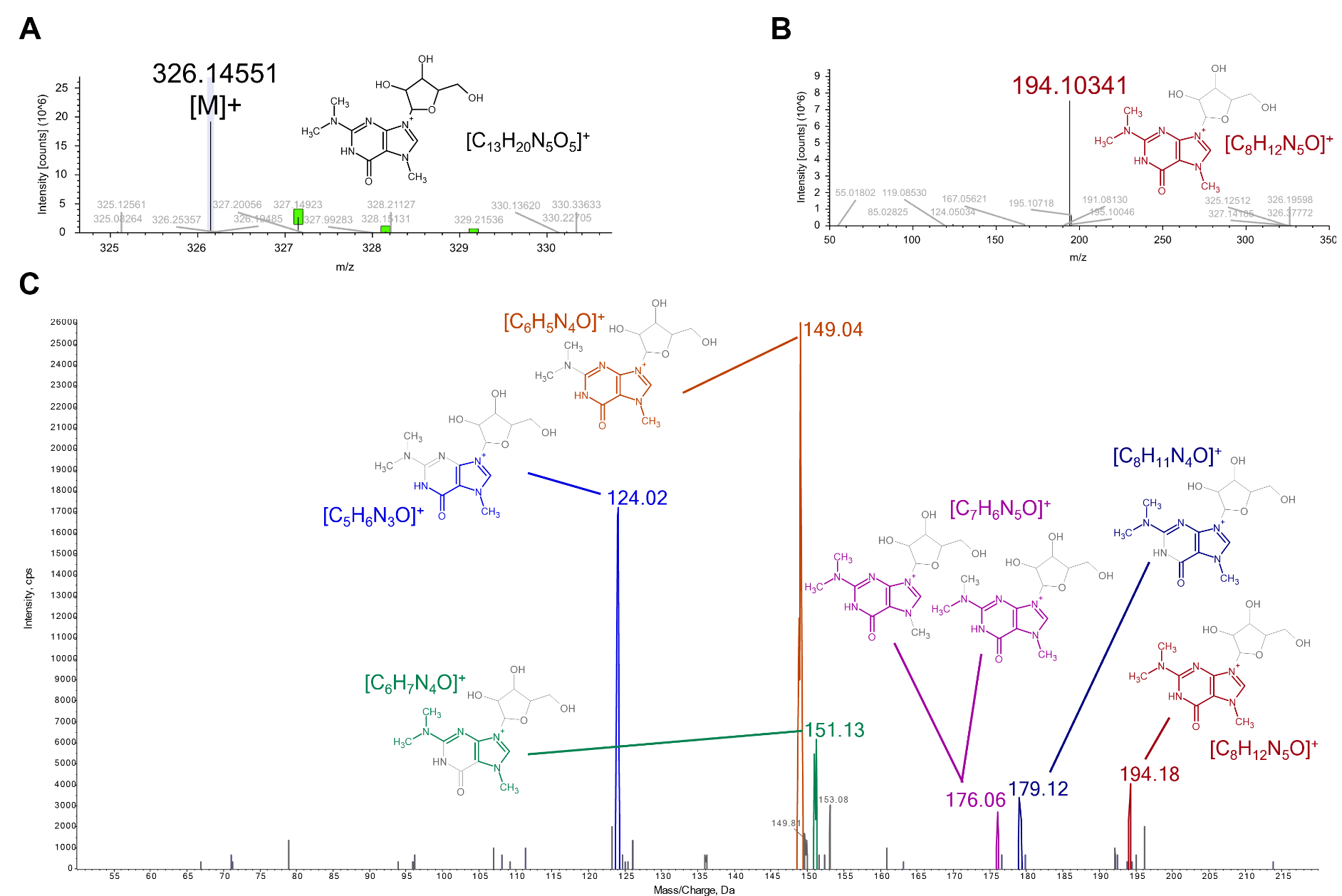


**Figure S2:** MS-data for structural elucidation of N2,N2,7-trimethylguanosine. (A) Molecular ion found in untargeted metabolomics analysis, (B) MS^2^-data found in untargeted metabolomics analysis and (C) fragmentation pattern of MS^2^-fragment with m/z 194.1 found in MS^3^ data.


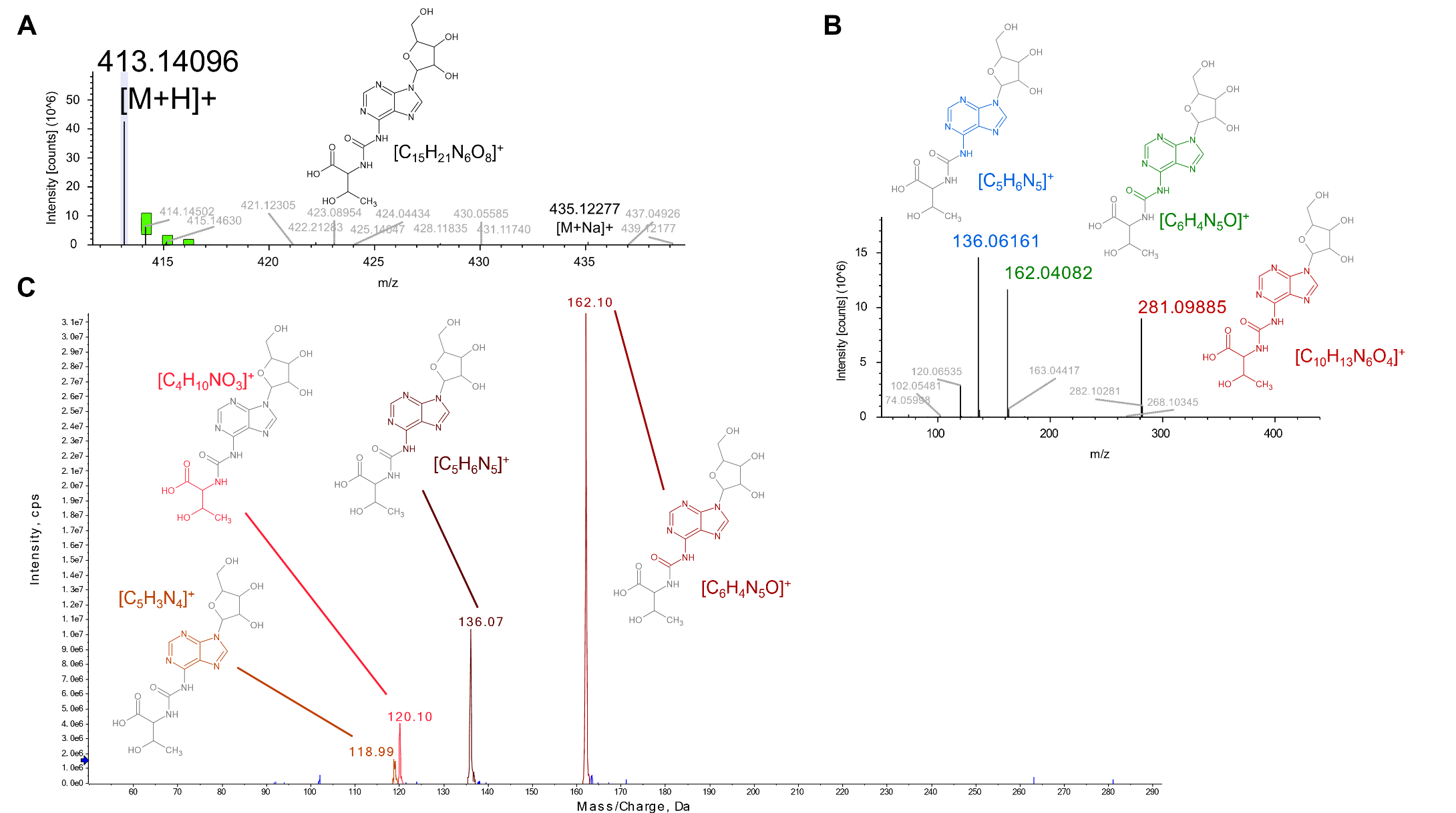


**Figure S3:** MS-data for structural elucidation of N6-threonylcarbamoyladenosine. (A) Molecular ions found in untargeted metabolomics analysis, (B) MS^2^-data found in untargeted metabolomics analysis and (C) fragmentation pattern of MS^2^ fragment with m/z 281.1 found in MS^3^ data.

**Figure S4:** Sequence effects on the change of N4-acetylcytidine in plasma found in the combined data of the DAPA and EMPA trials. Box plots indicate changes found after treatments in periods and sequences in comparison to previous time points or treatments as log_2_‑fold changes (log_2_fc). Whiskers of box plots show minimum and maximum log_2_fc.

**Table S1: Changes in plasma in the DAPA and EMPA trials**

| Compound | DAPA | | | EMPA | | |
| --- | --- | --- | --- | --- | --- | --- |
|  | log_2_fc | 95% CI | Adj. p-value | log_2_fc | 95% CI | Adj. p-value |
| 3-Hydroxybutyric acid | 0.73 | 0.25 to 1.52 | 0.045 | 0.94 | 0.41 to 1.54 | < 0.001 |
| 3 Hydroxybutyrylcarnitine | 0.70 | 0.36 to 1.24 | 0.043 | 0.36 | 0.10 to 1.24 | 0.016 |
| Uric acid | -0.23 | -0.35 to -0.08 | 0.005 | -0.23 | -0.32 to -0.09 | 0.003 |

Data of log_2_-fold changes (log_2_fc) are shown as the intraindividual median with the 95% confidence interval (CI) of the median. Respective p-values are calculated with paired t-tests and adjusted via Benjamini-Hochberg.
